# WormQTL^HD^—a web database for linking human disease to natural variation data in *C. elegans*

**DOI:** 10.1093/nar/gkt1044

**Published:** 2013-11-11

**Authors:** K. Joeri van der Velde, Mark de Haan, Konrad Zych, Danny Arends, L. Basten Snoek, Jan E. Kammenga, Ritsert C. Jansen, Morris A. Swertz, Yang Li

**Affiliations:** ^1^Genomics Coordination Center, University of Groningen, University Medical Center Groningen, P.O. Box 30001, 9700 RB Groningen, The Netherlands, ^2^Groningen Bioinformatics Center, University of Groningen, P.O. Box 11103, 9700 CC Groningen, The Netherlands, ^3^Department of Genetics, University of Groningen, University Medical Center Groningen, P.O. Box 30001, 9700 RB Groningen, The Netherlands, ^4^Department of Bioinformatics, Hanze University of Applied Sciences, Groningen, Zernikeplein 11, 9747 AS, The Netherlands and ^5^Laboratory of Nematology, Wageningen University, 6708 PB Wageningen, The Netherlands

## Abstract

Interactions between proteins are highly conserved across species. As a result, the molecular basis of multiple diseases affecting humans can be studied in model organisms that offer many alternative experimental opportunities. One such organism—*Caenorhabditis elegans*—has been used to produce much molecular quantitative genetics and systems biology data over the past decade. We present WormQTL^HD^ (Human Disease), a database that quantitatively and systematically links expression Quantitative Trait Loci (eQTL) findings in *C. elegans* to gene–disease associations in man. WormQTL^HD^, available online at http://www.wormqtl-hd.org, is a user-friendly set of tools to reveal functionally coherent, evolutionary conserved gene networks. These can be used to predict novel gene-to-gene associations and the functions of genes underlying the disease of interest. We created a new database that links *C*. e*legans* eQTL data sets to human diseases (34 337 gene–disease associations from OMIM, DGA, GWAS Central and NHGRI GWAS Catalogue) based on overlapping sets of orthologous genes associated to phenotypes in these two species. We utilized QTL results, high-throughput molecular phenotypes, classical phenotypes and genotype data covering different developmental stages and environments from WormQTL database. All software is available as open source, built on MOLGENIS and xQTL workbench.

## INTRODUCTION

Many exciting data sets have been collected in recent years for *Caenorhabditis elegans*, a free-living, non-parasitic soil-related nematode that feeds on the bacteria of decaying organic matter. This worm has many useful features that have made it one of the most studied model organisms*:* it is small and easy to house, has a short generation time and is transparent. As a consequence, its genomic information is now available (1), and the developmental path and function of almost every cell in its body has been described (2). In addition, recent genetical genomics studies in *C. elegans* have revealed thousands of genomic regions (loci) that are associated to the quantitative variation in a diverse range of phenotypes, such as gene expression [expression Quantitative Trait Loci (eQTLs)] (3–9), lifespan (10), development (11–13), stress resistance (14,15), behaviour (16,17), dauer formation (15,18) and sensitivity to RNAi treatments (19).

Genes having eQTLs mapping to the same genomic region (i.e. hotspot) are possibly involved in the same biological pathway/process. Palopoli *et al.* (5) have shown that biochemical processes and molecular functions of genes are generally highly conserved. Lee *et al.* (20) have shown that using the OMIM database (21) (http://omim.org/) and orthologue mapping data from INPARANOID (22), it is possible to infer new gene–gene interactions that are responsible for a certain disease in man from model organism data. McGary *et al.* (23) have shown that the conservation level between *C. elegans* and man is sufficient to infer gene–gene interactions in man from worm data. Even though the global disease phenotypes may not be at all comparable, the molecular basis may be common (e.g. breast cancer and high male incidence of progeny). For example, research on stress response in *C. elegans* has provided detailed insight into the genetic and molecular mechanisms underlying complex human diseases (24). In addition, Shaye and Greenwald (25) have generated a compendium of *C. elegans* genes with human orthologues using four orthology prediction programmes for identifying *C. elegans* orthologues of human disease genes for potential functional analysis. As a result, linking *C. elegans* and human data could help to understand the mechanisms underlying many human diseases.

To facilitate the exploitation of the worm eQTL data for human disease research we developed a new database, WormQTL^HD^, which quantitatively and systematically links many eQTLs findings in *C. elegans* to gene–disease associations in human. The database is based on the detection of the overlapping sets of orthologous genes associated with different phenotypes, or ‘phenologs’ (23) between these two species. The data, mainly eQTL results, were taken from different platforms (e.g. Agilent) and experiments (e.g. developmental stages). We provide a set of web-based analysis tools to search the database and explore phenotypes based on gene orthologues between worm and man. The result can be downloaded and visualized in a comprehensive yet clear way. All data and tools can be accessed via a public web user interface, as well as basic programming interfaces, which were built using the MOLGENIS biosoftware toolkit (26).

To our knowledge, this is the first online database for the systematic investigation of *C. elegans* phenotype equivalents of human diseases by integrating known disease–gene associations, gene orthologue data, molecular phenotypes and QTL results. WormQTL^HD^ allows researchers to explore these complex data in a user-friendly way, finding new genes, interactions and loci for human disease models.

WormQTL^HD^ is freely accessible without registration and is hosted at http://www.wormqtl-hd.org. All underlying software is open source and can be downloaded and freely used, for example, as a local mirror of the database and/or to host new studies, which can be uploaded using XGAP format (27). Below we describe the results, methods used to implement the system and future plans.

## IMPLEMENTATION

**Figure 1.**
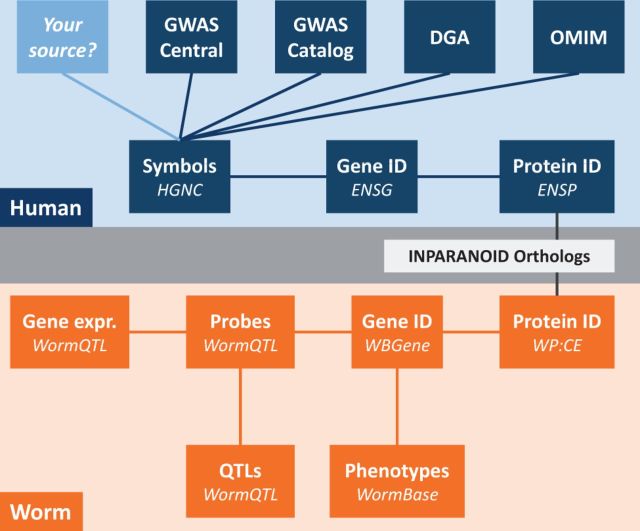
Human and worm data integration. WormQTL^HD^ was compiled using data derived from WormQTL, WormBase, OMIM, DGA, GWAS Catalogue and GWAS Central.

WormQTL^HD^ was compiled using data from six sources that are described below: (I) WormQTL (24,28), (II) WormBase Phenotypes (29), (III) Online Mendelian Inheritance in Man (OMIM) (21), (IV) The Disease and Gene Annotations (DGA) (30), (V) NHGRI GWAS Catalogue (31) (http://www.genome.gov/gwastudies) and (VI) GWAS Central (32,33) (Figure 1). (I) WormQTL (http://www.wormqtl.org) contains many published ‘genetical genomics’ experiments and consists of 47 public data sets with eQTL data on 500 panels (Recombinant Inbred Lines or natural strains), 68 452 microarray probes, 1630 samples and 1579 markers. The tools that were present in WormQTL, such as the QTL Finder and the Genome Browser, are also available in WormQTL^HD^. (II) WormBase is ‘an international consortium of biologists and computer scientists dedicated to providing the research community with accurate, current, accessible information concerning the genetics, genomics and biology of *C. elegans* and related nematodes’ (WormBase Mission statement, Todd Harris, 26 November 2012). From WormBase, we downloaded all the gene–phenotype associations (total 227 216) via WormMart. (III) OMIM is one of the most popular databases containing 14 164 human gene–disease associations. (IV) The DGA database (2961 associations) was started in 2013 and claims to be more comprehensive than OMIM. (V) The NHGRI GWAS Catalogue is a collection of 12 925 SNP-to-disease associations published in GWAS studies with at least 100 000 assayed SNPs and a *P*-value of <1.0 × 10^−^^5^. The SNPs were linked to human genes by the authors of the original papers that have been included in the catalogue. (VI) GWAS Central (32–34) is a database of summary level findings from genetic association studies. The authors of GWAS Central gathered and curated many datasets from public domain projects, and supplied us with a list of 4487 gene–disease associations having a *P*-value of <1.0 × 10^−^^10^. Because of the non-overlapping information in these four sources of human genes linked to disease, they are all provided and can be selected by the user. Human and worm data are connected based on the detection of orthologous genes in these two species. We downloaded all INPARANOID orthologues between *C. elegans* and *Homo sapiens* with a 100% bootstrap value. The bootstrap value indicates how often the pair is found as reciprocally best matched in a sampling with a replacement procedure that was applied to the original Blast alignment.

**Figure 2.**
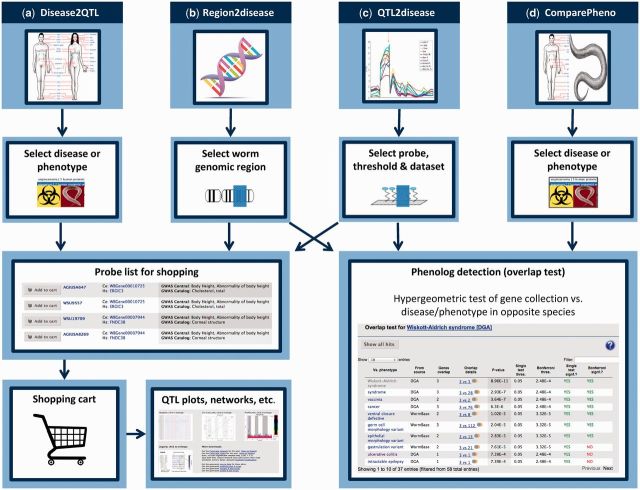
Cross-experiment search. WormQTL^HD^ provides four tools to explore the database: mapping human diseases to worm QTLs (Disease2QTL); mapping a worm genomic region to human diseases (Region2disease); mapping worm QTLs to human diseases (QTL2disease); and linking worm phenotypes to human diseases (ComparePheno).

To explore this database, WormQTL^HD^ features four major searching tools for different purposes. The starting points are summarized in Figure 2 and described in detail below, followed by a short summary of the software used.

### Tool 1: ‘Disease2QTL’, mapping human diseases to worm eQTLs

Exploring the genetic variation data for human gene orthologues in worm can provide useful insight into the function and regulation of human diseases. WormQTL^HD^ provides a tool for human geneticists to explore novel causal genes for a specific human disease by using worm QTL findings. Using a selection of one or multiple human diseases (from OMIM, DGA, NHGRI GWAS Catalogue or GWAS Central), a ‘shopping’ page is presented with worm gene expression probes and their human disease association. More information about the gene orthology mapping and association studies can be browsed. Users can put individual probes, or all probes at once, into the ‘shopping cart’. Subsequently, they can explore the genetic variation of those genes across the different experiments and studies that are stored in the WormQTL^HD^ database. The shopping cart is a central place in WormQTL^HD^ where users can see the various worm gene probes that they have selected, and create QTL/eQTL visualizations from the items in the shopping cart using ‘Plot QTLs’.

Using the ‘Plot QTLs’ function, researchers can test if genes associated with the selected diseases have any QTLs and if they map to a common genomic region. Shared QTLs suggest that those genes are regulated by the same genetic variation and are possibly involved in the same biological pathways. The genes with *cis-*QTLs in that genomic region are used as candidate genes in several types of studies (35–37). The same approach can be used for causal genes of human diseases. Alternatively, users can also select worm phenotypes (1504 total) instead of human diseases as a starting point. The shopping window is presented in exactly the same way as before, so users can browse human diseases from a worm phenotype perspective instead, or simply shop for probes of choice for a given worm phenotype and plot their QTLs, without considering any human disease relation.

### Tool 2: ‘Region2disease’, mapping worm genomic regions to human diseases

Researchers can link worm genomic regions to human diseases. This approach starts by selecting a region in the worm genome, e.g. a known ‘eQTL hotspot’, where a number of eQTLs are located. The region is selected by providing the chromosome name, start and end base pair positions. Users can quickly define a region of interest by using the location of any *C. elegans* gene. The database then returns all worm gene expression probes that are annotated in this region. From the probes, the corresponding worm genes are gathered, plus their human orthologues. The user is presented with a table containing the human-worm orthology and disease/phenotype associations in man and worm. After shopping for some or all of the relevant probes, users can choose to visualize eQTL results for them (similar to Tool 1), or perform a disease enrichment test.

The hypergeometric gene overlap test (38) to discover phenologs (phenotype orthologues) can be performed by clicking on ‘Disease enrichment’. All probes in the region are linked to their corresponding genes in worm, and a test is performed whether this entire group of genes is significantly ‘enriched’ for one or more human diseases by overlapping orthologous groups and worm and human genes. The statistical significance of phenologs (*P*-value) is listed in an output table. A significant result means that the input genomic region shares a significantly larger set of orthologous genes with a human disease than would be expected at random, even if the expressed phenotype in worm appears very different from the human disease phenotype (e.g. breast cancer and fertility). This tool can provide novel interpretation of genomic regions of interest.

### Tool 3: ‘QTL2disease’, mapping worm QTLs to human diseases

Researchers can start by selecting a QTL/eQTL in worm to find potential relationships with human diseases. We can select QTLs of interest based on three criteria: a selected experiment, a certain threshold for significance (LOD score) and a specific gene expression probe with a suspected QTL. If there is a QTL with a LOD score above the threshold, we automatically select the closest 50 probes on both sides of the highest peak marker. These probes are presented and available for browsing, shopping and plotting of QTLs, or can be the input for the disease enrichment test to find phenologs.

### Tool 4: ‘ComparePheno’, linking worm phenotypes to human diseases

WormQTL^HD^ also provides a tool that links human diseases to classical worm phenotypes (and *vice versa*) to discover phenologs in a systematic way. Users begin by selecting one or more human diseases and clicking on ‘Compare’. The genes associated with the selected disease are tested for enrichment against all sets of known associated genes for worm phenotypes. The result reveals functionally coherent, evolutionarily conserved gene networks.

Alternatively, users can also start by selecting worm phenotypes, which are tested against human diseases. In addition to cross-species testing, results of within-species disease enrichment are also available (e.g. to find the closest related human disease for another input human disease).

### Software used

All the software has been implemented using the open source ‘MOLecular GENetics Information Systems’—MOLGENIS—toolkit (26). The MOLGENIS toolkit is Java-based software to generate tailored research infrastructure on-demand (39). In particular, we built on an existing MOLGENIS application, the extensible xQTL workbench (40) and the R/qtl QTL mapping and visualization package for the R language (41,42). All software is available as open source on http://github.com/molgenis for others to reuse locally. Related technical documentation is available at http://www.xqtl.org, http://www.rqtl.org and http://www.molgenis.org.

## RESULTS

To demonstrate the added value of WormQTL^HD^, we have reproduced findings from known studies and have shown that novel insights and hypotheses can be achieved with little time and effort. Subsequently, we performed a broad-sweep disease-enrichment test to find all non-evident phenologs and to explore which new putative candidate genes for human diseases could be elucidated for future research.

### Case 1: Linking disease to worm phenotype from McGary *et al.* (23)

McGary *et al.* performed a phenolog mapping between the high incidence of male *C. elegans* progeny to human breast/ovarian cancers. Of 4649 total orthologues, McGary *et al.* reported 3 overlapping genes of 12 human disease-associated genes and 16 worm phenotype-associated genes—which is a significant enrichment (hypergeometric test *P*-value of ≤7.2 × 10^−^^6^). From the 13 worm phenotype-associated genes that were not overlapping, 9 had orthologues that had already been linked to breast cancer in the primary literature. They implicated the remaining four genes as new breast cancer candidates. We replicated these findings using the ComparePheno tool of WormQTL^HD^, searching for the WormBase phenotype ‘high incidence male progeny’. The first human disease among the results is ‘{Breast cancer, susceptibility to}, 114480 (3)’ from OMIM. Our tool reported 2 overlapping genes of 4 human disease-associated genes and 63 genes from the worm phenotype. This resulted in a *P*-value ≤1.4 × 10^−^^3^ (uncorrected). The second best human hit in the results is ‘malignant neoplasm of ovary’ from DGA. We found two overlapping genes of six ovarian cancer associated genes, resulting in a *P*-value ≤3.41 × 10^−^^3^ (uncorrected). ComparePheno also indicated enrichment of these categories. The *P*-values are ‘less significant’ than McGary *et al.* because (i) their definition of ‘high incidence male progeny’ included only 16 rather than 63 genes and (ii) they used an older INPARANOID version, so the overlap test was performed on a different orthologue mapping. Together, these results from our database do indeed replicate their findings. See Online Use Case 1 on the Help page to repeat this case.

### Case 2: Worm eQTL hotspot from two temperature expression data from Li *et al.* (43)

Li *et al.* (43) found an eQTL hotspot (77.56 Mb on chromosome V) on the worm genome in which genetic variation is associated with the expression of 66 genes, while these genes are located elsewhere on the genome. This indicates that these genes are possibly involved in the same biological process/pathway and potentially share a regulatory element. They may be physically located on the eQTL hotspot, which controls gene expression responding to different ambient temperatures. First, we used the Region2disease tool and input positions ChrV:15430739–16430739 (a non-cumulative 1 Mb region around the hotspot). We put all 931 probes located in this region in the shopping cart, and selected ‘Disease enrichment’. The best hit was ‘Response to antineoplastic agents’ (agents used in chemotherapeutic treatment of cancer) from GWAS Catalogue (*P*-value ≤4.92 × 10^−^^3^, uncorrected). For this hit, the associated human gene, *PPP2R5E,* is orthologous to WBGene00012348 (*pptr-1*) present in this region. The best WormBase hit is ‘thermotolerance increased’ (*P*-value ≤1.5 × 10^−^^2^, uncorrected), also via association with *pptr-1*. Padmanabhan *et al.* (44) showed that *pptr-1* is involved in regulating subcellular localization and transcriptional activity of the forkhead transcription factor *daf*-16. Rodriguez *et al.* (24) reviewed the role of heat stress response experiments in *C. elegans* for detecting human disease genes. They reported that *daf-16* in worms controls lifespan and stress response. In humans, the *daf-16* orthologue *FOXO3A* is associated with aging and prevalence of cancer (45). Using the Disease2QTL tool, a search for ‘Response to antineoplastic agents’ results in six probes for orthologues of *PPP2R5E* (WBGene00012348) and *ACOX3* (WBGene00019060). We selected them all and plotted the QTLs. This revealed a highly significant (LOD > 50) *cis*-eQTL for *pptr-1* in the Rockman *et al.* (9) dataset. Given all the evidence, we believe *pptr-1* might be an interesting candidate in the further development of a temperature-based *C. elegans* model for understanding human cancer and developing potential therapeutic drugs. Moreover, it shows that combining the ‘Region2disease’ and ‘Disease2QTL’ tolls can lead to an interesting hypothesis ready for experimental validation. See Online Use Case 2 on the Help page to reproduce this case.

### Case 3: Osmotic stress as a model for Bardet–Biedl syndrome from Rodriguez *et al.* (24)

Rodriguez *et al.* proposed hypertonic or osmotic stress in *C. elegans* as a model to study human diseases related to protein aggregation, such as Alzheimer’s and Parkinson’s. Hypertonic stress due to loss of water causes an intracellular ionic imbalance, which leads to rapid accumulation of organic osmotic glycerol and accumulation of damaged proteins. Shaye and Greenwald (25) showed that *osm-12* (associated with osmotic stress response) is orthologous to *BBS7* in man, which is associated to Bardet–Biedl syndrome (46). We used the Disease2QTL tool to look for QTLs associated with Bardet–Biedl syndrome by selecting all ‘Bardet-Biedl syndrome’ entries (seven in total) from OMIM. When we plotted the QTLs in worm for these entries, three significant eQTLs (LOD > 5) were found for *osm-12* (in *cis*), *bbs-5* (also in *cis*) and *bbs-2* (in *trans*). The strongest QTL (LOD > 6) was found for *bbs-5*, reported by probe AGIUSA3442 in the Rockman *et al.* dataset. We used the QTL2disease tool to investigate this QTL further. It revealed a nearby, very significant eQTL (LOD > 10) for a gene named *T07C4.10*, which can be investigated further as a potential candidate for this disease model. See Online Use Case 3 on the Help page to replicate this example.

### Novel disease–gene associations by ‘broad-sweep’ disease-enrichment test

We performed hypergeometric gene overlap tests to find phenologs between all worm phenotypes versus all human diseases. Table 1 lists the 15 most significant hits for human diseases that have significant gene overlap with worm phenotypes (see Supplementary Table S1 for the top 100). New candidate genes for human diseases can be discovered from phenologs by investigating human orthologues of worm genes that did not overlap with known human genes of the disease of interest.

**Table 1.** Top 15 results for the ‘broad-sweep’ disease enrichment

Phenotype_1_ (Ce)	Phenotype_2_ (Hs)	*n*_1_	*n*_2_	*k*	*P*-value
Peroxisome physiology variant	Zellweger syndrome, 214100 (3) (OMIM)	3	4	3	3.58E-10
Coenzyme Q depleted	Coenzyme Q10 deficiency, 607426 (3) (OMIM)	9	3	3	7.53E-09
Spontaneous mutation rate increased	Mismatch repair cancer syndrome, 276300 (3) (OMIM)	42	4	4	9.88E-09
Mitochondrial metabolism variant	Coenzyme Q10 deficiency, 607426 (3) (OMIM)	17	3	3	6.09E-08
AWA odorant chemotaxis defective	Cardiofaciocutaneous syndrome, 115150 (3) (OMIM)	3	2	2	3.64E-07
Peroxisome physiology variant	Adrenoleukodystrophy, neonatal, 202370 (3) (OMIM)	3	3	2	1.09E-06
AWC odorant chemotaxis defective	Cardiofaciocutaneous syndrome, 115150 (3) (OMIM)	5	2	2	1.21E-06
Germ nuclei rachis	Cardiofaciocutaneous syndrome, 115150 (3) (OMIM)	6	2	2	1.82E-06
Excretory cell development variant	Rheumatoid arthritis (GWAS Catalogue)	3	5	2	3.64E-06
Bacterially unswollen	Cardiofaciocutaneous syndrome, 115150 (3) (OMIM)	11	2	2	6.67E-06
Organism starvation response variant	Ovarian cancer, somatic, 604370 (3) (OMIM)	12	2	2	8.00E-06
Neuron development variant	Diastolic blood pressure (GWAS Catalogue)	17	11	3	9.85E-06
Ventral closure defective	Wiskott–Aldrich syndrome (DGA)	8	3	2	1.02E-05
Egg laying imipramine resistant	Bone mineral density (GWAS Catalogue)	26	23	4	1.08E-05
mRNA export variant	disease by infectious agent (DGA)	4	6	2	1.09E-05

*n*_1_ indicates the number of orthologues in *C. elegans* (Ce) with phenotype_1_, *n*_2_ the number in *H. sapiens* (Hs) with phenotype_2_ and *k* the number in both sets. The significance of each phenolog is assessed by the hypergeometric probability (*P*-value).

McGary *et al.* (23) reported ‘Zellweger syndrome’ in man to be a phenolog with ‘Reduced number of peroxisomes’ in yeast (*P*-value <1.0 × 10^−^^9^). Our best hit was ‘Zellweger syndrome’ with ‘peroxisome physiology variant’ in worm (*P*-value <3.6 × 10^−^^10^). Encouragingly, certain top hits such as ‘coenzyme Q depleted’ in worm versus ‘Coenzyme Q10 deficiency’ in man, and ‘spontaneous mutation rate increased’ in worm versus ‘Mismatch repair cancer syndrome’ in man make sense, thereby validating this approach and adding credibility to potentially non-evident human disease models.

## DISCUSSION

The current version of WormQTL^HD^ (August 2013) is a comprehensive and compendious database that enables molecular model organism data to be studied in the context of human diseases. Just as with WormQTL (24), we believe that WormQTL^HD^ will be continuously curated by the members of the *C. elegans* community. The results of the ‘broad-sweep’ disease-enrichment test in combination with the web tool will be of special interest to researchers in the human or worm domain. We believe these results could also be applied to prioritize the pathogenic variants increasingly being produced by next-generation sequencing in diagnostic labs. Genetic variants affecting human genes of unknown function may have worm orthologues that are part of human-worm phenologs and these may reveal or imply a role in a human disease. Thus, through functionally conserved networks, missing information can be inferred and candidate genes can be selected via model organisms.

The approach of WormQTL^HD^ is conceptually similar to that described by Smedley *et al.* (47). They created an automated method called PhenoDigm to provide evidence about gene–disease associations by analysing phenotypic information. In their case, phenotypes consist of a collection of ontology terms, which are aligned and scored to derive an overall phenotype-similarity score. Using this method, known gene–phenotype associations in model organisms (mouse, zebrafish) can be transferred to other organisms such as man, and help us to understand the genetic cause of disease. This method works best when the model organism is physiologically close to man and has comparable classical phenotypes. It would therefore be less useful for *C. elegans*. However, combining the molecular (WormQTL^HD^) and phenotypical (PhenoDigm) approaches may result in a very powerful tool to discover novel gene–disease associations in man, especially when using physiologically close model organisms.

We plan to further develop the WormQTL^HD^ data and toolset. There might be more ways in which researchers would like to search through the large amounts of data, for example, based on custom lists of gene identifiers, or by combining tools such as finding QTLs within specific regions. The QTL plots could be improved or replaced with interactive graphs that are more informative and would allow the users to continue ‘drilling down’ in the data instead of returning to the home page for a new analysis with a different tool. Furthermore, we envisage close integration with other data sources and tools such as WormNet, R/qtl and GO Enrichment to provide even more biological context and analytical tools for the user.

Our new database makes this data attractive and easy-to-use for an even wider community of quantitative geneticists working on worms and man. We are committed to maintaining the data and software in the future and invite the community to add and share their new data and ideas.

## SUPPLEMENTARY DATA

Supplementary Data are available at NAR Online.

## FUNDING

European Union Seventh Framework Programme (FP7/2007-2013) research projects BioSHaRE-EU [261433 to K.J.V. and M.A.S.]; Research Project PANACEA [222936 to J.E.K. and R.C.J.]; TI Food and Nutrition [TIFN GH001 to M.A.S.]; Dutch Carbohydrate Competence Center [CCC WP23 to K.Z.]; Centre for BioSystems Genomics (CBSG) and the Netherlands Consortium of Systems Biology (NCSB), both of which are part of the Netherlands Genomics Initiative/Netherlands Organisation for Scientific Research [to D.A.]; ERASysbio-plus ZonMW project GRAPPLE—Iterative modelling of gene regulatory interactions underlying stress, disease and ageing in *C. elegans* [90201066 to L.B.S.]; Netherlands Organisation for Scientific Research (NWO) VENI grant [863.13.011 to Y.L.]. Funding for open access charge: EU FP7.

*Conflict of interest statement.* None declared.
